# Children With Dyscalculia Show Hippocampal Hyperactivity During Symbolic Number Perception

**DOI:** 10.3389/fnhum.2021.687476

**Published:** 2021-07-20

**Authors:** Sertaç Üstün, Nazife Ayyıldız, Emre H. Kale, Öykü Mançe Çalışır, Pınar Uran, Özgür Öner, Sinan Olkun, Metehan Çiçek

**Affiliations:** ^1^Department of Physiology, Ankara University School of Medicine, Ankara, Turkey; ^2^Neuroscience and Neurotechnology Center of Excellence, Ankara, Turkey; ^3^Department of Interdisciplinary Neuroscience, Health Science Institute, Ankara University, Ankara, Turkey; ^4^Brain Research Center, Ankara University, Ankara, Turkey; ^5^Program of Counseling and Guidance, Department of Educational Sciences, Ankara University Faculty of Educational Sciences, Ankara, Turkey; ^6^Department of Child and Adolescent Psychiatry, Ankara University School of Medicine, Ankara, Turkey; ^7^Department of Child and Adolescent Psychiatry, Bahçeşehir University School of Medicine, İstanbul, Turkey; ^8^Department of Mathematics Education, Final International University, Kyrenia, Cyprus

**Keywords:** dyscalculia, functional magnetic resonance imaging, hippocampus, learning disabilities, number sense

## Abstract

Dyscalculia is a learning disability affecting the acquisition of arithmetical skills in children with normal intelligence and age-appropriate education. Two hypotheses attempt to explain the main cause of dyscalculia. The first hypothesis suggests that a problem with the core mechanisms of perceiving (non-symbolic) quantities is the cause of dyscalculia (core deficit hypothesis), while the alternative hypothesis suggests that dyscalculics have problems only with the processing of numerical symbols (access deficit hypothesis). In the present study, the symbolic and non-symbolic numerosity processing of typically developing children and children with dyscalculia were examined with functional magnetic resonance imaging (fMRI). Control (*n* = 15, mean age: 11.26) and dyscalculia (*n* = 12, mean age: 11.25) groups were determined using a wide-scale screening process. Participants performed a quantity comparison paradigm in the fMRI with two number conditions (dot and symbol comparison) and two difficulty levels (0.5 and 0.7 ratio). The results showed that the bilateral intraparietal sulcus (IPS), left dorsolateral prefrontal cortex (DLPFC) and left fusiform gyrus (so-called “number form area”) were activated for number perception as well as bilateral occipital and supplementary motor areas. The task difficulty engaged bilateral insular cortex, anterior cingulate cortex, IPS, and DLPFC activation. The dyscalculia group showed more activation in the left orbitofrontal cortex, left medial prefrontal cortex, and right anterior cingulate cortex than the control group. The dyscalculia group showed left hippocampus activation specifically for the symbolic condition. Increased left hippocampal and left-lateralized frontal network activation suggest increased executive and memory-based compensation mechanisms during symbolic processing for dyscalculics. Overall, our findings support the access deficit hypothesis as a neural basis for dyscalculia.

## Introduction

The ability to understand and manipulate numerosity is called “number sense” and is essential in perceiving our surroundings given that numbers are one of the most important features of modern life (Dehaene, 1997). Number sense and arithmetical abilities are one of the most critical predictors of economic and social success (Parsons and Bynner, 1997).

Number sense was once considered a language-dependent skill acquired through appropriate education. However, evolutionary and developmental studies have shown that number sense is an ability that has phylogenetic and ontogenetic origins; therefore, it has a biological basis (Ansari, 2008; Nieder and Dehaene, 2009). In other words, various animal species and infants have basic number sense ability and there are shared characteristics of number sense in humans and animals that suggest a phylogenetic continuity (Dehaene, 2001). One of these common features across species is known as the “distance effect,” which is a difficulty in comparing two numbers as the numerical distance between these two numerosities decreases. The other shared characteristic is called the “size effect” which indicates that performance on comparing two numbers decreases with increasing number size (Moyer and Landeauer, 1967). These two effects could be considered as results of the “ratio effect.” Comparison performance depends on the ratio between two numbers so as the numbers get larger, the distance between them should also increase for easy comparison in accordance with Weber’s Law. In other words, the ratio effect refers to that comparison difficulty depends on the ratio between the two quantities (Lyons et al., 2015; Hohol et al., 2020).

This innate ability to process quantities, which is independent of education or culture, is often referred as the “approximate number system” (ANS). The ANS (also known as the “core number system”) allows humans to represent quantities in an approximate manner (Odic and Starr, 2018). It allows non-symbolic quantities to be processed without the need for symbolic representations. However, it is insufficient for perceiving and processing numerosity in an exact manner. Exact processing of large quantities requires symbolic representations (Wynn, 1992; Feigenson et al., 2004). The ability to represent and process numerical symbols (e.g., Arabic numerals) is acquired in appropriate educational and cultural contexts (Zhang and Norman, 1995). The cognitive system that allows for the representation and processing of numerical symbols is called the “symbolic number system” (SNS). Various researchers have hypothesized that the ANS has a critical role as the foundation of the SNS (Dehaene, 1997; Feigenson et al., 2004, 2013), while others have suggested that these are two parallel but distinct systems (Lyons et al., 2012; Damarla and Just, 2013).

Besides the behavioral foundations summarized above, neuroanatomical correlates of number sense and dyscalculia are also an important focus in the area. The critical role of the parietal cortex, especially the intraparietal sulcus (IPS) in the neural representation of number sense, has been known for a long time since early lesion studies (Gerstmann, 1940; Cipolotti et al., 1991; Lemer et al., 2003). Neuroimaging studies support these findings and indicate the supramodal role of parietal areas in number processing regardless of cultural and notational differences (Pinel et al., 2001; Eger et al., 2003; Tang et al., 2006). Dehaene et al. (2003) offer the Triple-Code Model, which suggests a distributed network of domain-specific and domain-general areas for the neural correlates of number sense. According to this model, parietal regions have domain-specific roles that are related to numerical processing while prefrontal regions are responsible for domain-general processes such as memory, attention, and executive control. Nevertheless, some researchers argue that frontal areas are not only responsible for domain-general processes associated with numerical cognition but are also directly responsible for number sense itself. Two recent meta-analyses showed that frontal regions are activated in number tasks almost as consistently as parietal areas and emphasized the importance of the roles of both areas for number sense (Sokolowski et al., 2017; Arsalidou et al., 2018). As well as the frontoparietal network, occipitotemporal, and hippocampal areas are also found to have key roles in number processing, indicating a close relationship between number perception and recognition/memory processes (Peters and De Smedt, 2018).

In the number sense literature, it is known that neural correlates of the ANS (for non-symbolic quantities) and SNS (for symbolic quantities) differ. Although they share common areas of activation to some extent, brain networks that are responsible for symbolic and non-symbolic numerical stimuli have distinctions (Piazza et al., 2007; Holloway et al., 2010). Some studies have shown that symbolic numerosity tasks activate a left-lateralized network while right-lateralized brain areas are more related to non-symbolic tasks (Venkatraman et al., 2005; Mock et al., 2018).

Problems with numerical perception can cause difficulties in learning arithmetical skills. Developmental dyscalculia is a specific learning disability affecting the acquisition of numerical and arithmetical skills in children even though they have normal intelligence and age-appropriate school education (American Psychiatric Association, 2013). The prevalence rate of dyscalculia has often been found to be between 3.5 and 6.5% in epidemiological studies (Gross-Tsur et al., 1996; Shalev et al., 2000). A recent epidemiological study revealed that dyscalculia has a 5.7% prevalence rate (Morsanyi et al., 2018). It has been suggested that dyscalculia is a heterogeneous disorder because of the multiple factors underlying it (Träff et al., 2017). Moreover, there is a high degree of comorbidity between dyscalculia and other disorders such as dyslexia and attention deficit hyperactivity disorder (ADHD) (Wilson and Dehaene, 2007; Price and Ansari, 2013; Träff et al., 2017). In a prevalence study, 64% of children with dyscalculia were shown to have dyslexia (Lewis et al., 1994). Similarly, it has been found that 26% of dyscalculics have attention problems (Gross-Tsur et al., 1996). Another epidemiological study revealed that even though the prevalence rate of dyscalculia is 6%, pure dyscalculia cases without comorbidities account for 1.8% (von Aster and Shalev, 2007). Comorbidities, the heterogeneity of the disorder and divergent participant inclusion criteria make it difficult to achieve consistent results and cause slow overall progress in the field, which leads to inadequate diagnosis and treatment methods for dyscalculia (Price and Ansari, 2013).

There are two competing domain-specific hypotheses about the main cause of dyscalculia, which refer to the deficit caused by problems in the ANS and SNS systems. The first hypothesis suggests that the main deficit in dyscalculia is in the innate core number system (or ANS) (Butterworth, 1999, 2005; Dehaene, 2001). According to this hypothesis, the problem in the basic representation of numerosity will lead to failing in both non-symbolic and symbolic tasks since the problem would be at the core of number sense. Some behavioral studies have found that children with dyscalculia have difficulties in both non-symbolic and symbolic number tasks and argue that the problem with dyscalculia lies in core number systems (Landerl et al., 2004; Mussolin et al., 2010). The second hypothesis, called the “access deficit hypothesis,” suggests a deficit in the link between quantities and their symbolic representations (in other words, a connection problem between the ANS and SNS systems). Therefore, dyscalculics should have a problem with symbolic tasks while they have no problems with non-symbolic quantity processing (Wilson and Dehaene, 2007). Behavioral findings in favor of supporting the access deficit hypothesis indicated that individuals with dyscalculia only perform less well than typically developed groups in symbolic number tasks. Also, greater reaction times among the dyscalculia group in symbolic tasks are interpreted as being due to the use of different strategies taking more time to process symbols (Rousselle and Noël, 2007; De Smedt and Gilmore, 2011; Gómez-Velázquez et al., 2015).

Neuroimaging studies assessing children with developmental dyscalculia revealed some functional and morphometrical differences in brain networks related to number sense (Price and Ansari, 2013; Kucian and von Aster, 2015). Some studies found reduced activation in the frontoparietal network in the dyscalculia group (Kucian et al., 2006; Rotzer et al., 2009). On the other hand, there are studies that found increased activation in the frontoparietal network in dyscalculics. It is argued that increased brain activation is a result of compensatory mechanisms in the brain (Kaufmann et al., 2009).

The purpose of our study is to examine the neural basis of dyscalculia by using a homogeneous and non-clinical sample. Specifically, the neural mechanism of symbolic and non-symbolic quantity processing in typically developing children and children with dyscalculia is investigated to illuminate the root cause of dyscalculia. For this purpose, almost 2000 children were screened to avoid clinical bias and to reach a homogeneous sample of pure dyscalculia. Our experimental design was a visual quantity comparison task that included both dot array comparison and Arabic digit comparison to allow us to investigate the core number system and the symbolic representation system separately. The quantity comparison task is one of the most widely used tasks for examining numerical magnitude processing (Ansari et al., 2005; Cantlon et al., 2009). We used the functional magnetic resonance imaging (fMRI) technique to determine which brain areas are active during symbolic and non-symbolic quantity processing and how these activations differ between typically developing children and children with dyscalculia.

## Materials and Methods

### Participants

The recruitment of participants was conducted in two stages. In the first stage of the study, we screened 2058 third-grade students from 13 primary state schools. The schools were selected to represent low, medium and high socioeconomic backgrounds. Students were evaluated in terms of general and mathematical ability. Raven’s Standard Progressive Matrices Test (RPMT), which is made up of a series of diagrams with a missing part that completes the pattern, was used to evaluate general ability (Raven, 2000). The Mathematics Achievement Test (MAT) and Calculation Performance Test (CPT) are used for estimating mathematical ability. The MAT is designed for first to fourth graders and consists of questions about counting, number patterns, arithmetical problem solving, and fractions (Fidan, 2013). The CPT consists of five columns, each containing 40 questions on four operations, and one minute is given for each column (De Vos, 1992). Participants were divided into groups according to their ages (each group spanned 6 months) and the percentage distribution of each test was evaluated within the group to avoid the effect of age. Participants whose MAT and CPT scores were at the lowest 25th percentile were included in the second stage of the study as dyscalculia candidates. Likewise, participants whose MAT and CPT scores were between 35 and 75% were determined as a healthy control group. Being below the 10th percentile of the RPMT scores was the exclusion criterion for all participants.

In the second stage of the study, children were invited to undergo detailed neurocognitive evaluations 2 years after the first stage. First, they completed the Chapman and Chapman (1987) Handedness Inventory (its validity and reliability for use in the Turkish population were reported by Nalçacı et al., 2002), and left-handed participants were excluded. A child psychiatrist evaluated the children through the use of a semi-structured interview (Schedule for Affective Disorders and Schizophrenia for School Age Children-Present and Lifetime Version-Turkish Version), and determined children with comorbid disorders such as ADHD and anxiety disorder (Gökler et al., 2004). After the psychiatrist’s evaluation, the IQ levels of children were determined using the Wechsler Intelligence Scale for Children (WISC-R) by a psychologist. Children were also screened for reading ability to reveal a possible comorbidity of dyslexia. Reading 80 words per minute was the cut-off value for the reading test so that children who read less than 80 words per minute were excluded from the study (Öner et al., 2018). Also, MAT and CPT tests were repeated to determine whether dyscalculia was persistent. Children with comorbidities such as ADHD, dyslexia, and anxiety, and children who scored lower than 80 in verbal and performance subtests and overall IQ scores on the WISC-R, were excluded from the study.

Before the fMRI stage, children were familiarized with the MRI procedure with a training session inside a mock scanner, which was reported as the best alternative to sedation for reducing head movements (de Bie et al., 2010). Children who had no difficulties with the MRI environment were included in the fMRI stage of the study.

Despite our plans to reach a larger sample, we were able to reach a relatively small sample of participants especially for dyscalculia group due to strict exclusion criteria. However with these criteria we reached a homogeneous sample without any clinical bias. At the fMRI stage, five participants’ fMRI data were excluded due to head movement artifacts and technical problems. Ultimately, we were able to analyze fMRI data from 12 children with dyscalculia and 15 healthy controls. Table 1 shows demographic characteristics and scores on the numerical abilities and intelligence quotients of the participants.

**TABLE 1:** Demographic and psychometric profiles of the dyscalculia and control groups.

	Dyscalculia (mean/SD)	Control (mean/SD)	Test-statistics	*p*-Value
*N*	12	15		
Sex (F/M)	8/4	8/7	–	0.696^a^
Handedness (R/L)	13.75 (1.05)	14.40 (1.84)	77.00^b^	0.485
Age (years)	11.83 (0.58)^c^	11.79 (0.43)	0.17^c^	0.860

Numerical tests				

MAT	9.75 (3.08)	20.27 (2.58)	0.00^b^	<0.001
CPT sum	69.83 (16.42)	105.93 (10.94)	−6.65^c^	<0.001
CPT addition	19.25 (3.49)	25.60 (3.25)	13.00^b^	<0.001
CPT subtraction	15 (4.26)	20.60 (2.77)	−4.12^c^	0.003
CPT multiplication	14.25 (5.29)	21.53 (2.75)	−4.62^c^	<0.001
CPT division	8.25 (3.98)	16.48 (2.85)	6.00^b^	<0.001
CPT mix	13.08 (3.15)	20.73 (3.08)	−6.35^c^	<0.001

IQ tests				

WISC-R performance	99.5 (9.81)	103.07 (11.35)	−0.86^c^	0.398
WISC-R verbal	89.33 (8.51)	105.93 (10.5)	−4.43^c^	<0.001
Arithmetic sub test (from verbal IQ)	6.75 (2.01)	10.53 (3.04)	−3.70^c^	0.001
WISC-R total	93.58 (8.03)	105.20 (9.73)	36.50^b^	0.009
WISC-R total (corrected for arithmetic)	96.38 (SE: 8.03)	102.96 (SE: 2.44)^d^	2.58^d^	0.121
Reading evaluation	97.91 (15.04)	108.27 (25.73)	54.00^b^	0.080

*MAT, Mathematics Achievement Test, CPT, Calculation Performance Test, WISC-R, Wechsler Intelligence Scale for Children. ^*a*^Fischer’s exact test (two-sided). ^*b*^Mann–Whitney U test. ^*c*^Independent samples *t*-test. ^*d*^ANCOVA.*

The study was approved by the Ankara University Clinical Research Ethical Committee and the Ministry of National Education of Turkey. The children and their parents were informed about the study and written informed consent was obtained from the parents.

### Experimental Design

The present study aims to explore neural correlates of two controversial hypotheses regarding whether dyscalculia is related to a problem with the core number system or symbolic representation system. For this purpose, a visual task was designed that allowed us to investigate these two systems separately, using Psychtoolbox software, which runs *via* MATLAB (Mathworks, Sherborn, MA, United States).

**FIGURE 1:**
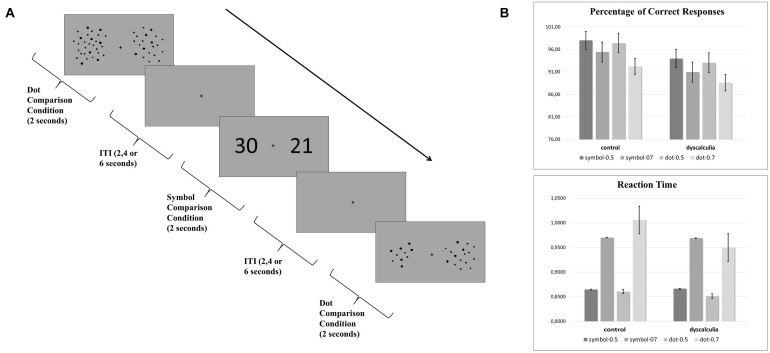
**(A)** Experimental quantity comparison paradigm that includes symbolic comparison and dot comparison condition. Each trial lasted for 2 s and intertrial intervals (ITI) were 2, 4, or 6 s. **(B)** Behavioral data. Graphs show the percentage of correct responses and reaction time results.

The experimental paradigm consisted of two different task conditions: symbol comparison (Arabic numerals) and dot comparison (array of dots) conditions. Each condition has two difficulty levels: an easy condition (0.5 quantity ratio, e.g., 25 vs 50) and a difficult condition (0.7 quantity ratio, e.g., 35 vs 50). In both conditions, two stimuli (black) were presented simultaneously with a gray background on the left and right sides of the screen (Figure 1A). Participants were asked to select the larger one by pressing the corresponding button. Participants performed the task while undergoing an fMRI scan.

The tasks were presented on a 28 × 37.5 cm screen with a distance of 72.5 cm from the participant’s eyes to the screen. The monitor resolution was 1024 × 768 pixels and the refresh rate was 60 Hz. In symbolic conditions, Arabic symbols were represented in 300-point font. Using non-symbolic quantities (array of dots) in number comparison tasks is especially challenging because changes in quantity correspond to the changes in spatial properties of the stimulus and it is found that these spatial differences could be used as clues for judgment of larger quantity and this could interfere with the results (Gebuis and Reynvoet, 2012). To prevent that we have tried to equate numerous visual properties in non-symbolic task. The size of dots, density and cumulative surface area of dot arrays varied across trials to prevent participants attending to the spatial features of dot arrays rather than numerical features. In half of the trials, the larger value had a greater surface area, while in the other half, the smaller value had a greater surface area. Therefore, participants could not use spatial information as a clue to judge numerical information.

An event-related fMRI design was used. There were four sessions during the fMRI acquisition and each lasted approximately 3 min 20 s. Each session included 36 trials and each trial lasted 2 s. There were 18 trials for each condition (symbol comparison and dot comparison) and 18 trials for each difficulty level (0.5 and 0.7 ratios), therefore there were nine trials for each condition-difficulty pair in each session (e.g., symbolic comparison with 0.5 ratio). A set of quantity pairs were pre-determined for each ratio and this same set is used for both symbolic and dot comparison conditions. All of these pre-determined quantities corresponded to double-digit numbers in the symbolic comparison condition. Trials were rendered in a randomized order and the intertrial intervals (with a crosshair fixation in the center of screen) between trials were 2, 4, and 6 s arranged in a pseudo-randomized and logarithmic manner favoring shorter durations.

### Image Acquisition

A 3 Tesla Siemens Magnetom Trio MRI scanner with a Siemens 16-channel head coil was used for image acquisition. High-resolution T1-weighted anatomical scans were obtained [Time to Repeat (TR): 2600, Time to Echo (TE): 3.02, Field of View (FOV): 256 mm, matrix: 256 × 256 and slice thickness: 1.00 mm]. Functional scans were acquired in the axial plane using 46 slices with a 3 mm width and a 0 mm gap (TR: 2500, TE: 28, Matrix: 64 × 64, FOV: 192 mm, voxel size: 3 × 3 × 3 mm). Slices were collected in an interleaved fashion.

Participants laid supine in the scanner with the response pad under their right hand. Participants’ heads were positioned inside the head coil and were immobilized with cushions. A PC running Psychtoolbox *via* MATLAB was used to display the task and to collect participants’ responses. The visual stimuli were displayed on an MRI-compatible screen that was visible *via* a mirror placed in the head coil. Earplugs were used to muffle the scanner noise.

There were four functional sessions, and each session consisted of 73 TRs. At the end of image acquisition, there were 292 fMRI scans and one anatomical scan for each participant.

### Image Analysis

Collected fMRI data were analyzed using SPM12 software (Wellcome Department of Cognitive Neurology, London, United Kingdom) run *via* MATLAB.

In the preprocessing, functional images were realigned to correct movement artifacts. The realigned data were co-registered with high-resolution anatomical T1 images to enable anatomical localization and spatially normalized to MNI coordinates. We used a pediatric template generated by the TOM toolbox (Template-O-Matic Toolbox^1^) for the mean age of participants in the normalization process (Wilke et al., 2008). Finally, spatial smoothing was applied using a Gaussian kernel [full width at half maximum (FWHM) = 9 mm]. The MotionFingerprint toolbox was used to detect head movement artifacts in addition to the preprocessing (Wilke, 2012). The toolbox is specifically designed for pediatric MRI and provides two sets of head movement data, i.e., total displacement (TD) and scan-to-scan displacement (STS), for each volume. These data sets were analyzed with SPSS and there were no significant differences for either TD (*p* = 0.123; *t* = −1.599) or STS (*p* = 0.447; *t* = −0.733) between the two groups. Therefore, one can safely assume that motion did not affect the results of group comparison.

The first level analysis was performed using General Linear Model (GLM). For each session four conditions (symbol 0.5, symbol 0.7, dot 0.5, and dot 0.7), six motion parameters and one session mean regressor constitute the design matrix of the GLM. Contrast images for different conditions were created after the beta values of the regressors estimated by averaging the four sessions.

At the second-level analysis, the data were analyzed by two-way repeated-measures analysis of variance (ANOVA) using SPM12. ANOVA analysis consisted of three factors each with two levels: 2 number (symbol and dot) × 2 difficulty (0.5 and 0.7) × 2 group (dyscalculia and control). The IQ scores and ages added as covariates to the fMRI group analysis.

Statistical results were shown at the *p* < 0.001 level, and using a cluster-extent threshold of *k* ≥ 54 corrected for multiple comparisons. To get the cluster extent needed for the desired correction for multiple comparisons at the *p* < 0.05 level, a Monte Carlo simulation was run with 10,000 iterations, using a type 1 error voxel activation probability of 0.001 (Slotnick, 2017, 2019).

A region of interest (ROI) analysis was performed to visualize group differences and interaction effects. We selected activated clusters *via* the MarsBaR toolbox and defined them as physiological ROIs (Brett et al., 2002). The mean percent signal change values of ROIs were extracted and graphed to enable a better understanding of the nature of group differences and interaction.

## Results

### Behavioral Data

Repeated-measures ANOVA was applied for the behavioral data analysis (separately for the percentage of correct responses and reaction times) using SPSS v.23 software. Bonferroni correction was applied for multiple comparisons.

The percentage of correct responses and reaction times were analyzed using a 2 (task: symbol/dot) × 2 (difficulty: 0.5 quantity ratio/0.7 quantity ratio) × 2 (group: dyscalculia/control) repeated-measures ANOVA. For the percentage of correct responses, the main effect of difficulty was significant [*F*(1,24) = 11.271, *p* = 0.003, η^2^ = 0.320], while the main effect of group (*p* = 0.188) and the main effect of task were not significant (*p* = 0.50). Also, group-number (*p* = 0.878), group-difficulty (*p* = 0.942), number-difficulty (*p* = 0.238), and group-number-difficulty (*p* = 0.719) interaction were not significant (Figure 1B). Similarly, for the reaction times, the main effect of difficulty was significant [*F*(1,24) = 126.579, *p* < 0.001, η^2^ = 0.841]. The main effect of group (*p* = 0.679) and the main effect of number (*p* = 0.972) were not significant. Also, group-number (*p* = 0.327), group-difficulty (*p* = 0.230), number-difficulty (*p* = 0.380), and group-number-difficulty (*p* = 0.284) interaction were not significant (Figure 1B).

### Functional Imaging Data

#### Main Effect of Number

**FIGURE 2:**
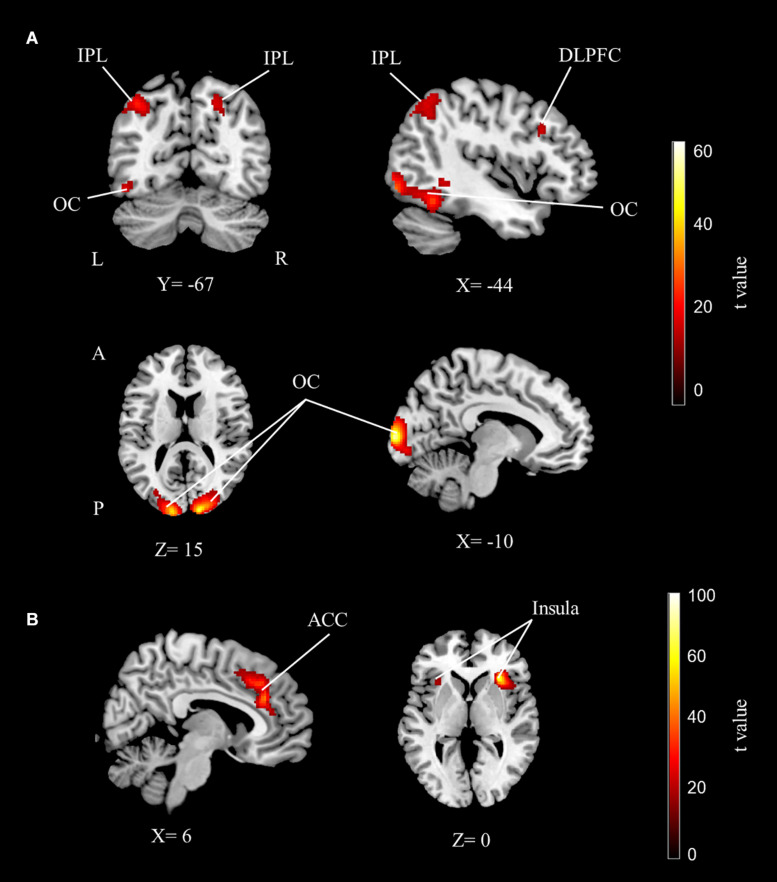
The fMRI group analysis results depicting significant activations. **(A)** The dorsolateral prefrontal, bilateral parietal, and occipital cortex activations related to the main effect of number sense. **(B)** The anterior cingulate and bilateral insular cortex activations related to the main effect of difficulty (cluster-extent corrected for *p* < 0.05 for all maps). IPL, inferior parietal lobe; DLPFC, dorsolateral prefrontal cortex; OC, occipital cortex; ACC, anterior cingulate cortex; L, left; R, right; A, anterior; P, posterior.

Results showed that while performing the numerosity tasks, the bilateral parietal cortices (although more extended in the left hemisphere), the left dorsolateral prefrontal cortex (DLPFC) and the left fusiform gyrus were significantly activated for both groups. In addition to these activations, the bilateral visual cortex, bilateral supplementary motor area, right peristriate cortex, and right posterior cingulate cortex were activated for the main effect of number (Table 2 and Figure 2A).

**TABLE 2:** Significant activations revealed by the ANOVA analysis (cluster-extent corrected for *p* < 0.05).

Brain region	Cluster size	Laterality	MNI coordinates			*Z*-score
			*X*	*Y*	*Z*	
**Main effect of number**						
Occipital cortex	2489	L	−8	−100	4	5.19
		R	14	−96	14	5.02
Fusiform gyrus	657	L	−46	−56	−18	4.35
Intraparietal sulcus	428	L	−38	−64	48	3.97
	173	R	20	−68	48	3.68
Supplementary motor area	152	R	6	−22	64	4.14
	71	L	−24	−20	48	4.44
Perisitriate cortex	117	R	44	−80	−6	4.28
Posterior cingulate cortex	95	R	6	−56	44	3.93
Dorsolateral prefrontal cortex	76	L	−46	20	32	3.89
**Main effect of difficulty**						
Insular cortex	1508	R	30	24	0	6.00
	158	L	−32	22	0	3.94
Anterior cingulate cortex	1380	Bilateral	2	26	40	5.38
	115	Bilateral	−2	6	28	4.57
Dorsolateral prefrontal cortex	734	R	38	50	24	4.51
Intraparietal sulcus	499	R	26	−60	36	4.29
	494	L	−20	−58	48	4.08
Cerebellum	213	L	−8	−78	−18	3.71
	90	L	−26	−66	−20	3.60
Inferior parietal lobe	192	R	48	−44	54	4.08
Posterior cingulate cortex	184	L	−2	−60	34	3.64
Occipital cortex	191	L	−28	−76	10	3.39
	100	R	18	−90	−4	3.51
	68	R	22	−68	6	3.81
Frontal eye field	84	L	−16	28	48	3.74
Premotor cortex	66	R	30	8	56	3.62
Fusiform gyrus	61	R	48	−48	−4	3.70
**Main effect of group**						
Anterior cingulate cortex	215	R	20	38	12	4.08
Medial prefrontal cortex	128	L	−6	52	28	3.78
Orbitofrontal cortex	62	L	−14	58	8	3.58
**Group–number interaction**						
Hippocampus	88	L	−16	−42	12	4.03

#### Main Effect of Difficulty

The right dominant bilateral insular cortex and anterior cingulate cortex were activated for the main effect of difficulty. The bilateral IPS, anterior prefrontal cortex, posterior cingulate cortex, frontal eye field, premotor cortex, fusiform gyrus, and cerebellum were also activated for difficulty (Table 2 and Figure 2B).

#### Main Effect of Group

**FIGURE 3:**
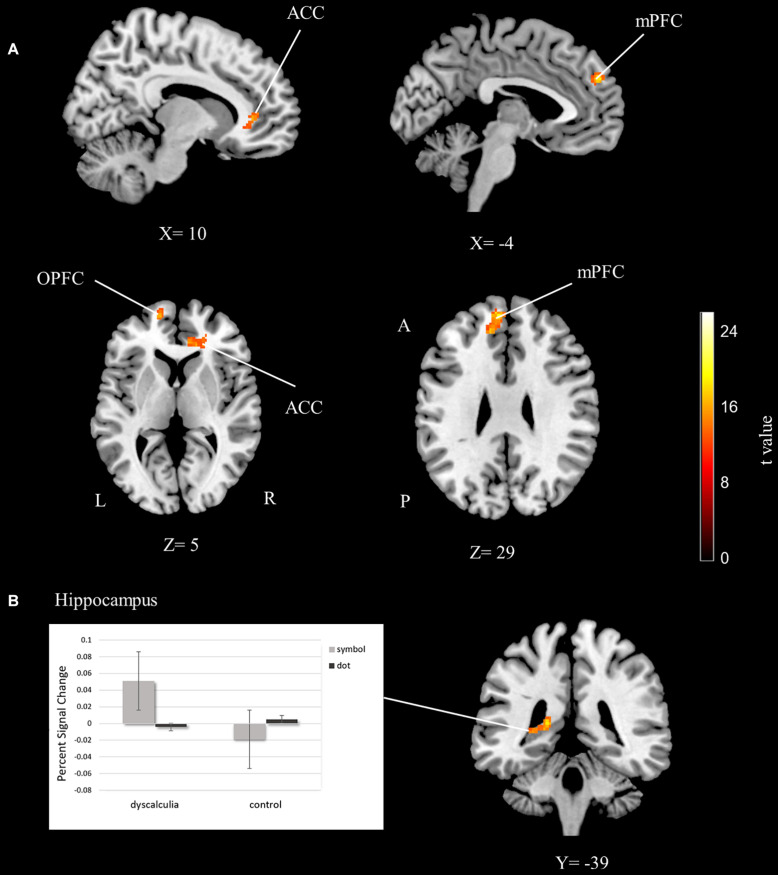
The fMRI group analysis results depicting significant activations. **(A)** The anterior cingulate, medial prefrontal, and orbitofrontal cortex activations related to the main effect of the group. **(B)** The hippocampus activations related to the group-task interaction. Percent signal change graphs that show differences of BOLD signal value extracted from hippocampus ROI (cluster-extent corrected for *p* < 0.05). ACC, anterior cingulate cortex; mPFC, medial prefrontal cortex; OPFC, orbitofrontal cortex; L, left; R, right; A, anterior; P, posterior.

Significant group differences were shown in the frontal areas. The orbitofrontal cortex, anterior cingulate cortex, and medial prefrontal cortex activations were found to be significant for the main effect of group (Table 2 and Figure 3A). Percent signal change was extracted from these areas and the data set is graphed. According to the percent signal changes, these areas showed higher activations in the dyscalculia group than in the control group (see Supplementary Figure 1).

#### Group-Number Interaction

The left hippocampus was significantly activated for group and number interaction (Table 2 and Figure 3B). In other words, activation differences between symbol and dot comparison conditions differ between groups. The percent signal change was extracted from the hippocampus activation to visualize this interaction and the data set is graphed. According to the percent signal changes, the hippocampus only showed higher activation in the dyscalculia group in the symbolic comparison condition but not in the dot comparison condition (Figure 3B).

#### Group-Difficulty Interaction

There was no significant activation in group and difficulty interaction. In other words, the distinction between the two difficulty levels was similar between the two groups.

## Discussion

In the present neuroimaging study, we investigated the neural basis of number sense and dyscalculia by using a non-clinical, homogeneous sample and a visual task that involved two different numerosity formats. In line with previous studies, we found frontoparietal network activations in all numerical processing tasks and mainly frontal activation differences in the dyscalculia compared to the control group. Also, our findings indicate that children with dyscalculia show more hippocampal activity during symbolic processing.

### Behavioral Findings

Behavioral results have shown no differences between dyscalculia and control groups. Adjusting the difficulty level of neuropsychological tasks is one of the main methodological difficulties in studies with dyscalculia groups (McCaskey et al., 2018). The difficulty level of our task was adjusted to avoid a ceiling effect for the control group and to ensure that the activation differences in the dyscalculia group were not caused by cognitive load.

There were also no significant differences between the two task conditions (symbolic comparison vs dot comparison). On the other hand, there was a significant difference between the two difficulty levels (0.5 and 0.7 quantity ratio), meaning a lower percentage of correct responses and higher reaction times in the difficult condition (0.7 quantity ratio). This finding is in line with the ratio effect phenomenon. As the numerical ratio between two numerosities decreases, the comparison of these two numbers gets more difficult and this can be observed in numerical symbols as well as non-symbolic quantities (Moyer and Landeauer, 1967; Moyer and Bayer, 1976; Dehaene, 2001; Lyons et al., 2015).

### Activations Related to Number Perception

The numerical task activations are in line with previous reports about number sense. Firstly, our results revealed bilateral IPS activation, which has long been known to be associated with numerical processing (Gerstmann, 1940). The Triple-Code Model has suggested that this region is a domain-specific region (Dehaene et al., 2003). Numerous neuroimaging studies in the field have found that IPS is related to numerical perception regardless of the notation (Eger et al., 2003; Ansari et al., 2005; Tang et al., 2006; Holloway and Ansari, 2010; Park et al., 2013). Our findings also revealed DLPFC activation during numerosity judgment. Similarly to the parietal cortex, the majority of studies in the field found prefrontal activations. Single cell recordings in primate studies revealed that intraparietal areas were responsible for numerical processing along with the prefrontal areas (Nieder and Miller, 2004; Nieder, 2012; Viswanathan and Nieder, 2013). It has been argued that even though the prefrontal cortex has domain-general roles (Ansari et al., 2005; Cantlon et al., 2006), it is also directly related to number sense (Nieder and Dehaene, 2009; Sokolowski et al., 2017).

Fusiform gyrus activation was also found for the main effect of number. It has been argued that the fusiform gyrus is related to number sense along with the well-known face and object recognition functions (Proverbio et al., 2018). Researchers suggest that a number form area in the fusiform gyrus is specifically related to numerical symbols (Shum et al., 2013; Amalric and Dehaene, 2016; Grotheer et al., 2016; Vatansever et al., 2020). A recent meta-analysis suggests that the number form area is a part of the numerical processing network including prefrontal and parietal areas (Yeo et al., 2017). Our findings also revealed extended bilateral visual area activation for number sense. Processing the array of dots requires visual efforts and therefore resulted in the activation of occipital areas (Born and Bradley, 2005). Directing attention to dots in order to compare them could cause occipital activation in different tasks (Üstün et al., 2017) as well as numerical tasks (Holloway et al., 2010).

In the present study, the main effect of difficulty, which has been examined by using the ratio effect, revealed mainly insular and anterior cingulate cortex activations. It has been shown that insular cortex activation increases as task difficulty increases, so this region is suggested to be related to task difficulty (Lamichhane et al., 2016). Ansari et al. (2005) showed that insular cortex activation was related to the difficulty levels of numerosity comparison. Anterior cingulate cortex activation is suggested to be responsible for executive control, working memory and attention processes that are related to numerical perception along with the prefrontal cortex (Mock et al., 2018). Number tasks usually require executive control, attentional resources and internal motivation, especially in children. Therefore insular activation in studies that involve children could be related to internal motivation while the anterior cingulate cortex along with the frontal eye field are responsible for the attentional mechanism (Arsalidou and Taylor, 2011; Vossel et al., 2014; Arsalidou et al., 2018).

### Activation Differences in Dyscalculia

In the present study, the main effect of group analysis revealed the activations in the anterior cingulate cortex, medial prefrontal cortex, and orbitofrontal cortex. Percent signal change graphs of these activations showed that these three regions have a higher level of activation in the dyscalculia group than in the control group (see Supplementary Material).

It has been argued that higher frontal activations in dyscalculics are the result of increased reliance on supporting executive functions as compensatory mechanisms (Kaufmann et al., 2009; Kucian et al., 2011). In line with this compensatory mechanism suggestion, a stronger connectivity between frontal and parietal regions was also reported in dyscalculics (Rosenberg-Lee et al., 2015). The higher anterior cingulate cortex activation in the dyscalculia group in the present study could be related to the increased need for attentional mechanisms, which is consistent with previous studies. It is argued that the increased anterior cingulate and frontal activation in children compared to adults occurs as a result of the higher attention and executive control needs of numerical processing at a young age (Ansari et al., 2005; Rivera et al., 2005; Cantlon et al., 2006). Similarly, children with dyscalculia could require more executive functions than healthy controls to overcome their difficulty in processing numerical quantities and this might be the reason for frontal and anterior cingulate activations in this study. In addition to the executive control, studies have shown prefrontal activations for symbolic processing and argued that the prefrontal regions are related to matching quantities with their symbolic representations (Cantlon et al., 2009; Holloway and Ansari, 2010; Mock et al., 2018). In light of these reports, the increased prefrontal activations in the dyscalculia group in our findings can also be interpreted as an indicator of a difficulty in symbolic processing.

Another main finding of our analysis was left hippocampal activation in task-group interaction. As revealed by the percent signal change graph, the hippocampus activation increased in the dyscalculia group and this increased activity occurred only in the symbolic comparison condition. Hippocampal activation was reported to be involved in numerical processing (Moeller et al., 2015). Moreover, the connectivity between the hippocampus and frontal regions was found to increase with improvement of numerical abilities (Supekar et al., 2013; Qin et al., 2014). Another study shows that hippocampal activation during numerical tasks is higher in children than in adults, indicating that children rely more on memory processes during numerical tasks (Rivera et al., 2005). It was also reported that adults with lower math achievement require higher hippocampus activation on a numerosity task (Haist et al., 2015). Critically, hippocampal activation in the present study was only observed during symbolic comparison in the dyscalculia group. Thus, this finding can be interpreted as children with dyscalculia resorting to memory-based compensatory mechanisms during symbolic processing to overcome the challenge. In other words, children with dyscalculia use their memory more than controls to match quantities with their symbolic representations in order to compare numbers written in symbolic format.

Numerous behavioral studies report difficulties in symbolic processing in dyscalculics These studies show that dyscalculics have lower scores than the control group in symbolic number tasks while they have the same scores as the control group in non-symbolic number tasks (Rousselle and Noël, 2007; De Smedt and Gilmore, 2011; Lafay et al., 2017). Rousselle and Noël (2007) also found a reduced distance and size effect during symbolic comparison in children with dyscalculia and they argued that this finding may indicate that children with dyscalculia use different strategies to process symbols. Memory-based strategies are suggested to be one of the methods that people use to compare numbers (Tzelgov et al., 1992). Based on these implications, it can be concluded that children with dyscalculia resort to different strategies to process symbols and that using memory mechanisms could be one of these strategies.

Overall, the presented results suggest that children with dyscalculia need different strategies to overcome a challenge during symbolic number processing. The previous findings suggested a left-lateralized frontoparietal network engagement during symbolic processing (Venkatraman et al., 2005; Piazza et al., 2007; Sokolowski et al., 2017). Increased left hemisphere hippocampal and frontal brain network engagement supports our view proposing a symbolic processing deficit in dyscalculia.

### Limitations

A small sample size could be regarded as the main limitation of the study. Despite a wide-scale screening, there was a big loss of participants in the MRI scanning stage of the study for external reasons. There was no difference between groups for total IQ score when it was corrected for arithmetic and it was added as a covariate to the fMRI analysis. However, different verbal IQ scores between groups could be a limitation of the study. Also, considerable difference in variance ratios of reading scores of the two groups could be regarded as a limitation for the appropriate group matching (Kover and Atwood, 2013). Lastly, to avoid movement artifacts we tried to keep the time that children spent in the fMRI scanner short, therefore relatively low trial numbers were obtained.

## Conclusion

The present study used a homogeneous non-clinical sample of pure dyscalculics to investigate the neural basis of the disease. Our findings showed a frontoparietal network activation (more extended on the left hemisphere) related to numerical processing together with the left fusiform gyrus (number form area). Left-lateralized frontal network activations in dyscalculics might be related to compensatory executive control and attention mechanisms. Our main finding was the left hippocampal activation found in dyscalculics (only in the symbolic comparison condition), which suggests that dyscalculics might use memory-based strategies as compensatory mechanisms to overcome symbolic processing difficulty. Overall, the present findings provide the first neuroimaging evidence supporting the access deficit hypothesis as the neural basis of dyscalculia.

## Data Availability Statement

The raw data supporting the conclusions of this article will be made available by the authors, without undue reservation.

## Ethics Statement

The studies involving human participants were reviewed and approved by the Ankara University Clinical Research Ethical Committee. Written informed consent to participate in this study was provided by the participants’ legal guardian/next of kin.

## Author Contributions

SÜ: methodology, investigation, formal analysis, and writing—original draft. NA: methodology, investigation, and writing—review and editing. EK: methodology, software, and investigation. ÖM, PU, and ÖÖ: investigation. SO: conceptualization, supervision, and writing—review and editing. MÇ: conceptualization, project administration, funding acquisition, supervision, and writing—review and editing. All authors contributed to the article and approved the submitted version.

## Conflict of Interest

The authors declare that the research was conducted in the absence of any commercial or financial relationships that could be construed as a potential conflict of interest.

## Footnotes

1 http://www.neuro.uni-jena.de/software/tom/
