# Supplementary material for: Children With Dyscalculia Show Hippocampal Hyperactivity During Symbolic Number Perception
Source: Front Hum Neurosci. 2021 Jul 20;15:687476. doi: 10.3389/fnhum.2021.687476 (PMC8330842; doi:10.3389/fnhum.2021.687476)
Supplement: Supplementary file 1 [file Data_Sheet_1.DOCX]

## Supplementary Material


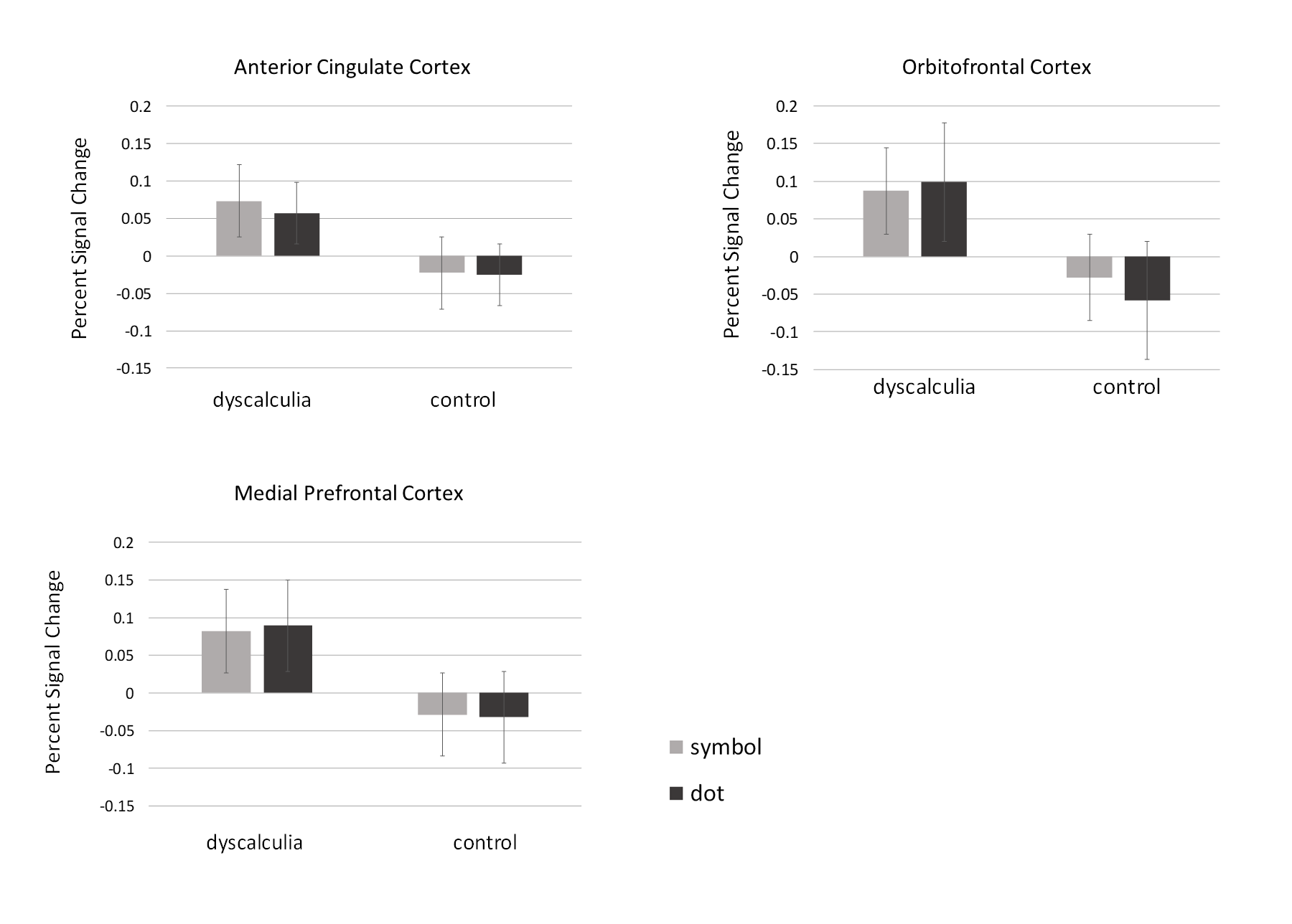


Figure S1. Percent signal change values of anterior cingulate, orbitofrontal and medial prefrontal cortex ROIs in dyscalculia and control
